# Pollen record of climate change during the last deglaciation from the eastern Tibetan Plateau

**DOI:** 10.1371/journal.pone.0232803

**Published:** 2020-05-06

**Authors:** Wei Shi, Hanchao Jiang, Xue Mao, Hongyan Xu

**Affiliations:** 1 State Key Laboratory of Earthquake Dynamics, Institute of Geology, China Earthquake Administration, Beijing, China; 2 College of Earth Sciences, University of Chinese Academy of Sciences, Beijing, China; Indiana University-Purdue University Indianapolis, UNITED STATES

## Abstract

The eastern Tibetan Plateau (TP) is a climatically sensitive area affected by the Indian Summer Monsoon (ISM). A new pollen record from a lacustrine sediment in Mao County shows that the study area was covered mainly by shrubs and herbs during the last deglaciation, indicating open and sparse forest grasslands. Hydrophilous herbs were mainly dominated by Cyperaceae, Poaceae, *Myriophyllum*, *Polygonum* and *Typha*, and they gradually increased from 18.7 to 16.8 ka, suggesting a transition to a more humid climate. This corresponds to climate cooling over the same period. From 16.8 to 14.6 ka, hydrophilous herbs continued to increase, coincident with a general ameliorating trend indicated by δ^18^O records from East Asia. Between 14.6 and 14.0 ka, the mean content of hydrophilous herbs reached peak in the sequence, corresponding to relatively high δ^18^O values during this period. From ~14.0 to 12.9 ka, the abundance of hydrophilous herbs decreased significantly. Over the same period, the Greenland ice core shows a decrease in δ^18^O and low-latitude cave stalagmites in China record an increase in δ^18^O. This implies that longitudinal temperature gradients increased and drove the southward retreat of the ISM, which in turn drove a continuous decrease in the abundance of hydrophilous herbs in the study area. From 12.9 to 11.6 ka, the mean content of hydrophilous herbs decreased to the lowest (8.3%) in the whole sequence, indicating a cold and dry climate in the study area. A positive shift in δ^18^O records during 11.6–10.6 ka was matched by a significant increase in the abundance of hydrophilous herbs in the study area, indicating a warm and humid climate trending. Hence, the ISM has had a significant impact on the climate of the eastern TP since the onset of deglaciation around ~16.8 ka.

## 1. Introduction

**Fig 1:**
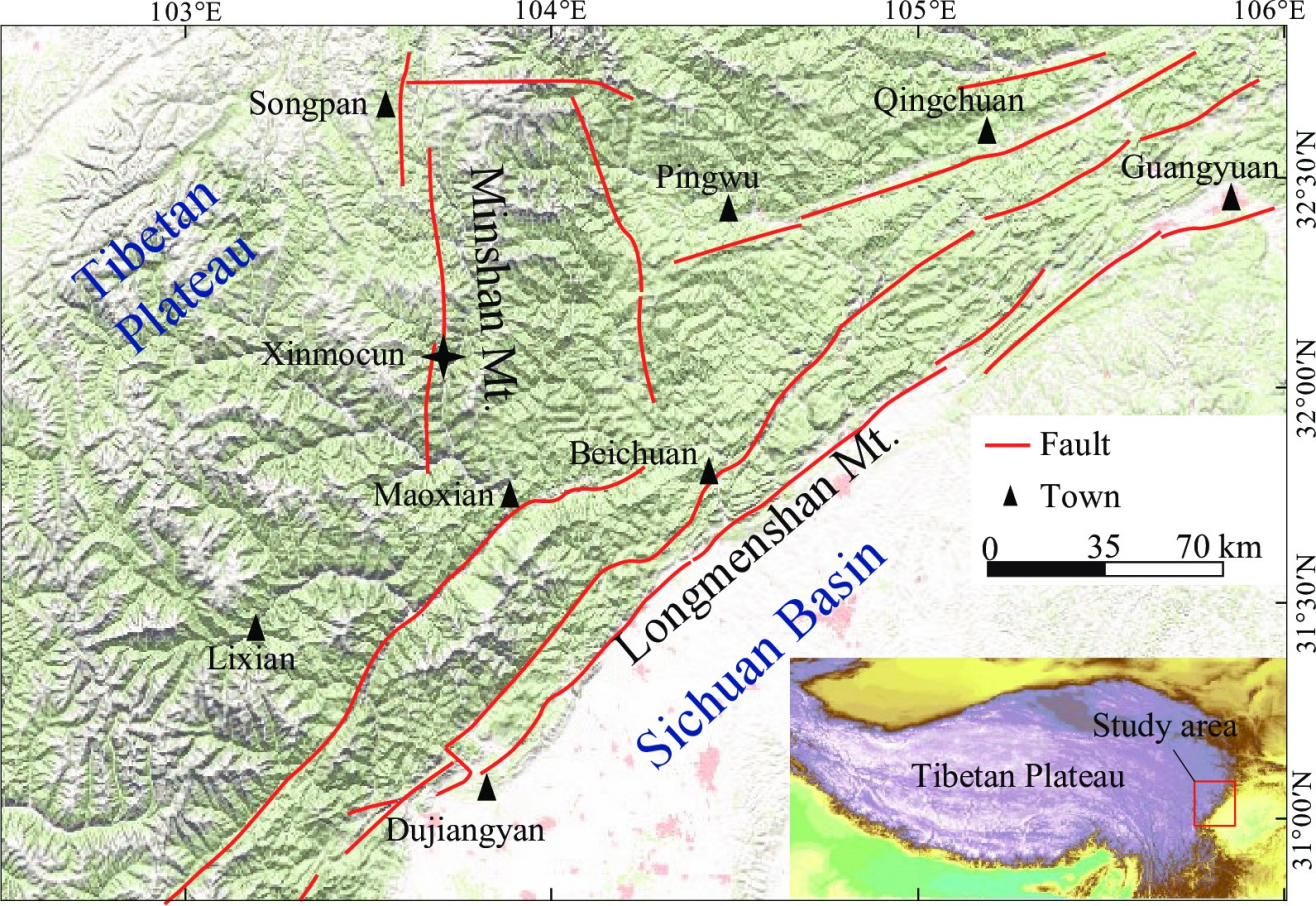
DEM of the eastern margin of the Tibetan Plateau illustrating the geomorphology and drainage systems (https://viewer.nationalmap.gov/advanced-viewer/). The star denotes the location of the Xinmocun lacustrine section.

The Cenozoic collision of the Indian and Asian plates resulted in uplift of the Tibetan Plateau (TP) and the formation of a steep eastern margin of the plateau, where the elevation decreases from >5000 m to ~600 m over a distance of ~50 km [Fig 1]. This transitional zone is characterized by frequent tectonic activity [1,2] and was settled by humans for agriculture relatively early [3,4], making it an interesting region for paleoclimatic and paleoenvironmental studies.

Recent observations of spatiotemporal patterns in precipitation have indicated that the climate of the eastern TP is dominated by the Indian summer monsoon (ISM) [Fig 1] [5–8]. Decadal/multi-decadal temperature discrepancy analysis along the eastern TP has suggested that long-term temperature and precipitation trends are coupled over the northern part of the eastern TP, but are decoupled over the southern part, and that the Zoige Plateau and adjacent regions represent a zone of dynamic climate that separates the northern and southern parts of the eastern TP [9]. Intriguingly, a speleothem record from the middle Yangtze region covering the last deglacial period recently indicates a wetter central eastern China during North Atlantic cooling episodes [10]. However, Wang et al. [11] argue that the East Asian Summer Monsoon activity increases with warming-wetting and decreases with cooling-drying. In addition, previous studies concentrated mainly on climate change during the Holocene period and have reported only low-resolution or obscure trends of climate change during the last deglaciation [6, 12–15]. Therefore, the main factors that affected the climate of the eastern TP during the last deglaciation remain unclear.

Pollen sequences are regarded as one of the most reliable continental archives of paleoclimate and paleoenvironment. Deposition occurs as pollen rain that can be characterized by the percentage of pollen components; i.e., the pollen spectrum. This spectrum provides a reliable record of regional vegetation and therefore regional climate [16,17].

The last deglaciation was a critical transitional period from the Last Glacial Maximum to the beginning of the Holocene. In this study, we investigate the well-dated Xinmocun lacustrine section to reveal the climatic conditions of the last deglaciation by analyzing variations in the pollen record. This record is correlated with other contemporary records from the eastern Asia, and the controlling mechanism of climate change during this period is addressed.

## 2. Geologic and geographic settings

**Fig 2:**
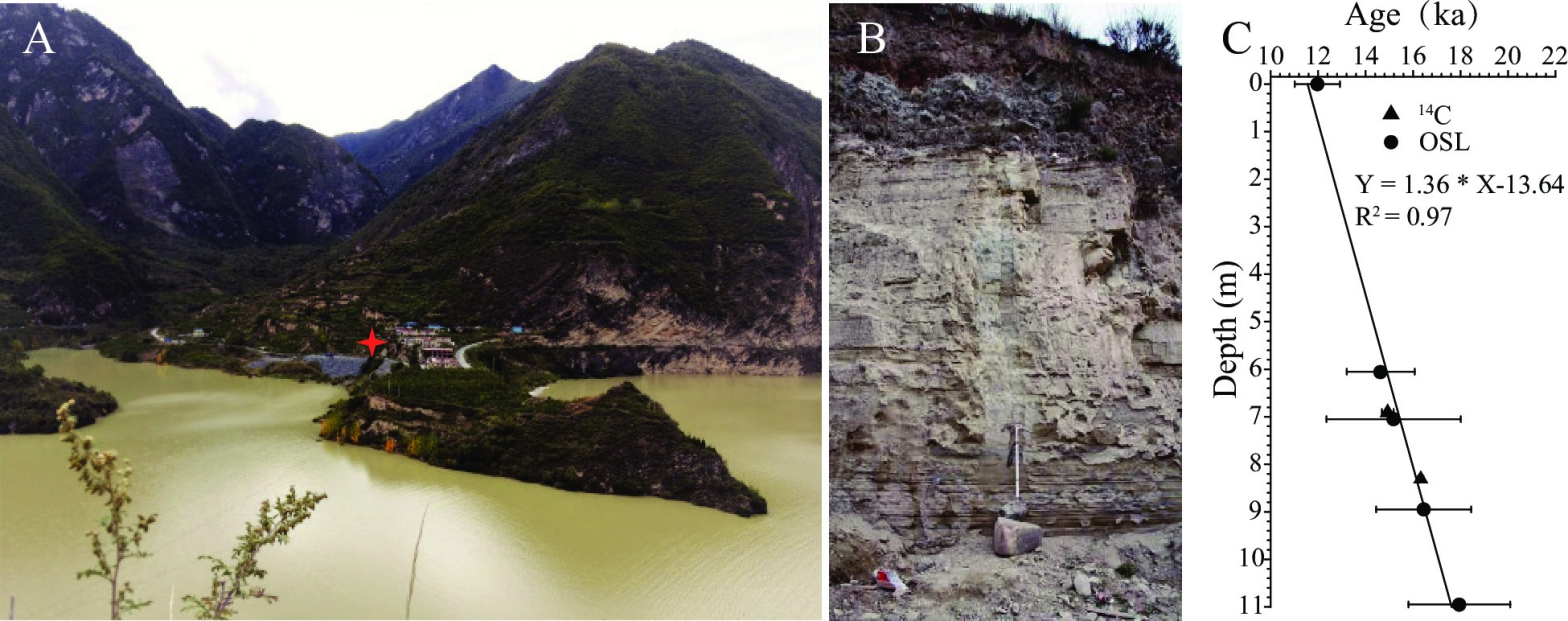
(A) Photograph showing the geomorphic features of the intermountain basins in the Diexi lake region and (B) the Xinmocun lacustrine section (red star). (C) ^14^C and OSL age versus stratigraphic depth of the Xinmocun section.

The Xinmocun lacustrine section (32°2.7′N, 103°40.1′E; 2188 m above sea level) is ~11 m thick and is located in an intermountain basin at Diexi on the eastern TP [Fig 2A] [18]. The Diexi lake remains a lake today and our samples were collected from an outcrop well exposed in the field. No specific permissions were required for these locations and field studies did not involve endangered or protected species. The landscape of the basin is characterized by alpine valleys dominated by two major mountain ranges: the Min Shan and Longmen Shan. The study area varies in elevation from 870 m to 6253 m. The valleys along the Min River are usually steep and narrow, with an incision depth of 800–3000 m [Fig 2A].

The climate in the study area varies from subtropical to cold-temperate with increasing latitude, and from arid in the Diexi intermountain basin to humid in the high mountains. The Diexi intermountain basin is a sub-frigid, semi-humid highland with an overall dry and windy climate, cool summers, and cold winters. The mean annual temperature at the Maoxian meteorological station (31.41°N, 103.51°E, 1590.1 m above sea level) is 11.2°C and the mean annual precipitation is 490.7 mm, with 70%–90% falling between June and September. The mean annual latent evaporation is 1375.7 mm, which is 2.8 times the annual precipitation [18–20].

The vegetation cover in the upper reaches of the Minjing River includes *Abies faxoniana*, *Picea asperata*, *Pinus densata*, and *Tsuga chinensis* according to the Vegetation Map of China [21]. The vertical zonation of vegetation changes distinctly with increasing elevation: 1) arid valley scrubs (1300–2200 m a.s.l.), dominated by *Caryopteris mongholica*, *Ajania potaninii*, *Leptodermis*, and *Caryopteris incana*, etc. 2) Mixed evergreen and deciduous broad-leaved forests (1600–2200 m a.s.l.), dominated by Lauraceae, *Quercus*, and *Acer*. 3) Mountain conifers (2000–3600 m a.s.l.), dominated by *Tsuga*, *Picea*, *Pinus*, *Abies*, and *Quercus aquifolioides*. 4) Alpine shrubs and meadows (> 3600 m a.s.l.), dominated by *Kobresia setchwanensis*, *Kobresia capillifolia*, and *Polygonum* etc. The economic trees are mainly *Juglans regia*, *Prunus salicina*, *Malus pumila*, and *Zanthoxylum bungeanum* [21]. A dataset analysis of the surface pollen on the TP indicates that the pollen buried in the sediments have the potential to reflect the intensity of the Asian Summer Monsoon at that time and provide a simple and potentially practical proxy for the study of the evolutionary history of the Asian summer monsoon [22].

## 3. Material and methods

The Xinmocun lacustrine sediments are dominated by silt (~76.6%) and have an average median particle size (Md) of 10.5 μm [18], making them suitable for pollen analysis [Fig 2B]. Pollen samples were collected from the section at intervals of 20 cm. Approximately 80 g of each subsample was processed following the newly revised palynological procedures described by Xu et al. [23], including treatments with 15% HCl and 3% NaOH, drying for 7–8 h in an oven at 85°C, heavy liquid separation with a KI solution (specific gravity, 1.74–1.76), treatment with 40% HF and 15% HCl, and sieving (7 μm) if necessary. All samples were centrifuged and washed with distilled water after each step. The prepared specimens were mounted in glycerol for identification. All samples were processed at the State Key Laboratory of Earthquake Dynamics, Institute of Geology, China Earthquake Administration (CEA).

## 4. Pollen record

AMS ^14^C dating of black carbon grains and optically-stimulated luminescence (OSL) dating of quartz grains have different signal sources and dating principles. Applying both methods to one section helps to test each other in reliability of dating results and then to better restrain the chronology of the section. Two new AMS ^14^C dates (S1 Table) and five published OSL dates [18] (S2 Table) show a good age-depth linear relationship (R^2^ = 0.97) and reveal a high average sedimentation rate of 1.37 mm/yr [Fig 2C], making it beneficial to study high frequency environmental changes in the eastern TP [18, 24].

**Fig 3:**
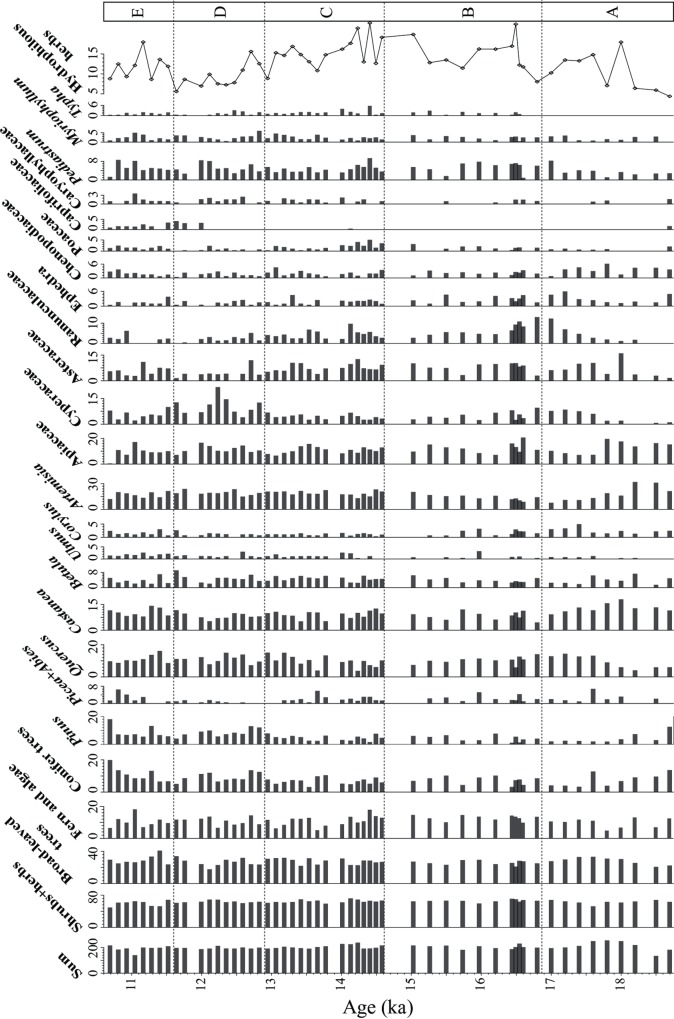
Pollen percentage diagram for the Xinmocun sediments on the eastern margin of Tibetan Plateau.

The age of each pollen sample was derived by linear interpolation of seven age dates [Fig 2C]. Fifty-three samples were analyzed and 12,772 spore and pollen grains were identified [Fig 3]. The fern spore and algae varied from 10 to 42 grains with a mean of 25 grains while the terrestrial pollen varied from 135 to 255 grains with an average of 203 grains. Fifty-nine taxa were identified, and these show a generally stable varying feature. To get better knowledge about terrestrial vegetation changes, all terrestrial pollen taxa were used to calculate the percentage of each pollen while all pollen and spore taxa were used to calculate the percentage of spores. Shrub and herb pollen (49.5%–72.1%, mean 63.8%) was dominant in all samples, comprising mainly *Artemisia*, Apiaceae, Cyperaceae, and Compositae. Among the taxa identified, Cyperaceae, Poaceae, *Polygonum*, *Typha*, and *Myriophyllum* were indicative of a humid environment [25–29, 30]; thus, the total percentage of these taxa was used as a proxy for relative humidity [Fig 3]. Broad-leaved pollen taxa (17.7%–40.9%, mean 27.4%) were dominated by *Quercus* (mean 10.2%) and *Castanea* (mean 9.7%), with minor *Betula*, *Ulmus*, *Corylus*, *Carpinus*, *Castanopsis*, *Alnus*, *Liquidambar*, *Pterocarya*, *Juglans*, and *Tilia*. Fern spores and algae (4.9%–18.1%, mean 11.0%) were dominated by *Polypodium* and *Pediastrum*. Conifer pollen (3.1%–19.9%, mean 8.1%) included *Pinus*, *Picea*, and *Abies*. The above taxa reflected an open and sparse forest grassland that covered the study area during the last deglacial period, which was generally consistent with the results of previous studies [31]. Five pollen zones were identified based on variations in the abundance of the dominant and hydrophilous pollen taxa [Fig 3].

### Zone A (9 samples, 18.7–16.8 ka)

Shrub and herb pollen in zone A (52.4%–68.2%, mean 63.1%) were dominated by *Artemisia* (7.7%–32.0%, mean 18.0%), Apiaceae (7.1%–19.6%, mean 12.8%), hydrophilous herbs (4.4%–18.0%, mean 10.4%), and Compositae (0%–8.5%, mean 3.9%) [Fig 3]. The former two taxa showed a relatively high abundance during the early part of the zone, and the latter two taxa showed a relatively high abundance during the late part. Other less dominant shrubs and herbs included Ranunculaceae (0%–12.3%, mean 3.5%), *Ephedra* (1.2%–6.0%, mean 3.0%), and Chenopodiaceae (1.0%–8.2%, mean 4.8%). Sporadically present taxa in this zone included Poaceae, Caprifoliaceae, *Thalictrum*, Lamiaceae, *Polygonum*, *Myriophllum*, and Caryophyllaceae. Notably, hydrophilous herb pollen (4.4%–18.0%, mean 10.4%) was dominated by Cyperaceae (1.6%–16.0%, mean 6.8%) and showed a clear increasing trend over time. Cyperaceae reached 16.0% at 18.0 ka, the highest value in the sequence [Fig 3].

Broad-leaved pollen (20.7%–33.2%, mean 28.2%) was dominated by *Quercus* and *Castanea*. The former increased from 4.1% to 14.4% over time with a mean of 9.4%, and the latter attained a relatively high value at the middle of the zone. *Betula* ranged from 1.5% to 7.3% with a mean of 3.9%. *Corylus* and *Ulmus* were frequently present, but in low amounts. *Liquidambar* occurred occasionally. Conifer pollen (3.3%–13.7%, mean 7.5%) was dominated by *Pinus* pollen (2.0%–12.6%, mean 4.1%) and showed a decreasing trend over time. *Picea* and *Abies* were present throughout the zone but in low abundance.

Fern spores and algae varied in abundance from 4.9% to 13.1% with a mean of 9.7%, showing relatively little variation over time [Fig 3]. They were dominated by *Pediastrum* (1.1%–8.3%, mean 3.6%) and monolete spores (0.5%–6.7%, mean 3.4%), along with *Polypodium* (0%–2.5%, mean 1.0%) and trilete spores (0%–4.8%, mean 1.3%). *Conentricystis* was sporadically present.

### Zone B (11 samples, 16.8–14.6 ka)

In this zone, the shrub and herb pollen count (59.5%–71.1%, mean 66.1%) was slightly higher than in zone A [Fig 3]. Both *Artemisia* (8.9%–20.4%, mean 14.1%) and Apiaceae (7.2%–20.7%, mean 12.4%) showed a clear increase over time, along with hydrophilous herb pollen (8.0%–22.5%, mean 14.7%). Cyperaceae was a dominant type, the abundance varied from 3.0% to 10.2% with a mean of 8.0%. Poaceae (0%–4.2%, mean 1.8%), *Polygonum* (0%–5.3%, mean 1.4%), *Myriophyllum* (0.4%–3.7%, mean 2.3%), and *Typha* (0%–2.9%, mean 1.2%) were frequently present but in low abundance [Fig 3]. Despite a clear increase in hydrophilous herb abundance, Cyperaceae decreased significantly between 15.8 and 15.2 ka, with the lowest value at 15.7 ka. Other commonly present shrubs and herbs included Compositae (2.4%–9.5%, mean 4.9%), Chenopodiaceae (1.4%–4.4%, mean 2.9%), Ranunculaceae (2.8%–13.1%, mean 6.9%), Thalictrum (0%-1.9%, mean 0.8%), and *Ephedra* (1.0%–4.7%, mean 2.7%). Caryophyllaceae and Lamiaceae occurred sporadically.

Broad-leaved tree pollen (21.1%–29.5%, mean 26.0%) showed a small decrease throughout this zone. *Quercus* (5.9%–14.1%, mean 10.3%), *Castanea* (4.5%–12.1%, mean 8.4%), and *Betula* (2.7%–6.5%, mean 3.9%) were less variable than in zone A. *Ulmus*, *Corylus*, *Liquidambar*, and *Juglans* occurred at low and highly variable percentages. *Pinus* pollen (1.1%–7.7%, mean 4.2%) was dominant over *Abies* and *Picea* (0%–5.2%, mean 2.2%).

Fern spores and algae (9.8%–14.6%, mean 12.8%) showed a slight increase in this zone [Fig 3], dominated by *Pediastrum* (0.9%–7.8%, mean 5.5%) and monolete spores (2.7%–6.7%, mean 4.5%). *Polypodium* (0%–2.1%, mean 0.9%) and trilete spores (0%–4.0%, mean 1.6%) were present with low mean values of < 2.0%. *Concentricystis* was occasionally present.

### Zone C (15 samples, 14.6–12.9 ka)

In zone C, shrub and herb pollen (59.0%–72.1%, mean 64.9%) was dominated by *Artemisia* (13.0%–23.2%, mean 19.0%), hydrophilous herbs (8.9%–22.8%, mean 15.5%), Apiaceae (6.6%–15.7%, mean 10.9%), Compositae (2.6%–6.8%, mean 4.3%), and Ranunculaceae (2.4%–9.7%, mean 4.4%), Chenopodiaceae (0%–6.1%, mean 2.6%), and *Ephedra* (0%–4.5%, mean 1.7%) [Fig 3]. Other taxa such as Caprifoliaceae, *Thalictrum*, Lamiaceae, Spiraeoideae, and Caryophyllaceae were present in a few samples (< 2.0%). Hydrophilous herb pollen (8.9%–22.8%, mean 15.5%) were mainly Cyperaceae (3.9%–12.6%, mean7.7%), Poaceae (0%–6.7%, mean 2.7%), *Polygonum* (0%–2.5%, mean 0.8%), *Myriophyllum* (0.8%–4.6%, mean 2.4%), and *Typha* (0%–5.7%, mean 1.8%), showing generally high values but decrease in abundance over time.

Broad-leaved tree pollen (22.1%–32.0%, mean 27.8%) was relatively stable over time and was dominated by *Quercus* (3.8%–15.1%, mean 9.7%), *Castanea* (5.2%–12.6%, mean 9.2%), and *Betula* (2.5%–6.3%, mean 4.7%), with lesser *Ulmus* (0%–3.5%, mean 1.4%) and *Corylus* (0.5%–2.2%, mean 1.5%). *Liquidambar* and *Juglans* were sporadically present. Conifer pollen (3.1%–10.5%, mean 6.8%) was dominated by *Pinus* (1.6%–7.8%, mean 4.6%). *Abies* and *Picea* pollen (0%–5.9%, mean 1.9%) was present at low and highly variable values.

Fern spores and algae were dominated by *Pediastrum* (3.2%–9.4%, mean 4.8%) and monolete spores (0.5%–7.7%, mean 3.8%), and they decreased through time from 17.9% to 5.1% with a mean of 10.9%. *Polypodium* (0%–1.6%, mean 0.6%) and trilete spores (0%–3.7%, mean 1.7%) also occurred.

### Zone D (10 samples, 12.9–11.6 ka)

Shrub and herb pollen (60.2%–69.3%, mean 63.9%) showed relatively little variation in zone D and was dominated by *Artemisia* (14.9%–23.9%, 19.3%), Apiaceae (7.1%–16.6%, mean 11.5%), and Compositae (4.0%–21.6%, mean 10.6%). Intriguingly, the mean value of Compositae in this zone was the highest of the entire section. In contrast, hydrophilous herb pollen (5.6%–15.6%, mean 9.3%) showed the lowest mean value of the section. The abundance of Cyperaceae pollen varied between 0% and 12.0%, with the lowest mean value (4.1%) of the entire section. Poaceae (0%–3.1%, mean 0.9%), *Polygonum* (0%–2.1%, mean 0.3%), *Myriophyllum* (0.5%–6.0%, mean 2.8%), and *Typha* (0%–3.1%, mean 1.2%) were regularly present in this zone. Chenopodiaceae (0.5%–3.8%, mean 2.0%), Ranunculaceae (0%–5.2%, mean 2.1%), Caryophyllaceae (0%–2.5%, mean 1.1%), and *Ephedra* (0%–2.5%, mean 1.2%) were present throughout, but at low abundances (< 2%). Other taxa occurred sporadically, including Caprifoliaceae, *Thalictrum*, Lamiaceae, and Spiraeoideae.

Relative to zone C, broad-leaved pollen in this zone (17.7%–34.0%, mean 26.3%) showed a slight decrease and were dominated by *Quercus* (7.3%–15.0%, mean 11.0%), *Castanea* (5.2%–11.7%, mean 8.4%), and *Betula* (2.1%–9.1%, mean 4.9%). *Ulmus* (0%–4.0%, mean 1.6%) and *Corylus* (0%–3.6%, mean 1.5%) pollen were present throughout the zone, although at low abundances. *Liquidambar* and *Juglans* occurred sporadically. Conifer pollen (5.1%–13.5%, mean 9.4%), dominated by *Pinus* (4.1%–13.0%, mean 8.4%), showed an increase in this zone. *Picea* and *Abies* pollen were locally present.

The abundance of fern spores and algae varied between 6.6% and 14.3% with a mean of 10.8%, and were dominated by *Pediastrum* (2.7%–8.5%, mean 5.1%) and monolete spores (0.9%–4.9%, mean 2.7%). Trilete spores (0%–3.3%, mean 2.1%) were present and *Concentricystis* occurred sporadically [Fig 3].

### Zone E (8 samples, 11.6–10.6 ka)

Shrub and herb pollen (49.5%–68.6%, mean 59.4%) decreased slightly in zone E relative to zone D. *Artemisia* (12.0%–21.4%, mean 17.0%), Apiaceae (0.5%–17.1%, mean 9.3%), Compositae (2.1%–10.0%, mean 5.6%), and hydrophilous herb pollen (8.6%–18.0%, mean 11.9%) were the main components. Cyperaceae (2.9%–11.0%, mean 5.9%) was the dominant hydrophilous herb, along with minor Poaceae (1.0%–3.3%, mean 2.1%), *Myriophyllum* (0.9%–5.0%, mean 2.4%), *Polygonum* (0%–1.4%, mean 0.2%), and *Typha* (0.5%–2.0%, mean 1.2%). Some other shrub and herb taxa were present but at low abundance, such as Chenopodiaceae (1.0%–4.9%, mean 2.6%), Caprifoliaceae (0%–3.3%, mean 1.6%), Ranunculaceae (0%–6.2%, mean 1.9%), *Thalictrum* (0%–2.1%, mean 0.8%), Lamiaceae (0%–1.0%, mean 0.3%), Spiraeoideae (0%–1.6%, mean 1.2%), Caryophyllaceae (0%–3.6%, mean 1.3%), and *Ephedra* (0%–3.8%, mean 1.4%) [Fig 3].

Broad-leaved pollen (23.8%–40.9%, mean 29.3%) showed an increase relative to zone D and was dominated by *Quercus* (8.6%–16.2%, mean 11.0%), *Castanea* (8.0%–14.2%, mean 10.4%), and *Betula* (2.0%–7.1%, mean 3.6%). *Ulmus* (1.5%–3.5%, mean 2.3%) and *Corylus* (1.0%–4.0%, mean 2.2%) remained present but at low abundances. *Liquidambar* and *Juglans* occurred sporadically. Conifer pollen (6.6%–19.9%, mean 11.0%) was dominated by *Pinus* (5.5%–18.1%, mean 8.7%). *Abies* and *Picea* pollen were present but at highly variable abundances [Fig 3].

Fern spores and algae (6.5%–18.1%, mean 10.4%) showed minor variations and were dominated by *Pediastrum* (1.3%–8.6%, mean 5.2%) and monolete spores (1.4%–2.9%, mean 2.3%). *Polypodium* and trilete spores were sporadically present [Fig 3].

## 5. Discussion

The Xinmocun palynoflora was dominated by shrubs and herbs with lesser arboreal pollen and fern spores, and algae. Among the shrub and herb pollen, the hydrophilous herbs, dominated by Cyperaceae, Poaceae, *Myriophyllum*, *Polygonum*, and *Typha*, showed clear changes in abundance during the last deglacial period, consistent with previously published records and factors that controlled the environmental conditions, as discussed below.

### 18.7–16.8 ka

**Fig 4:**
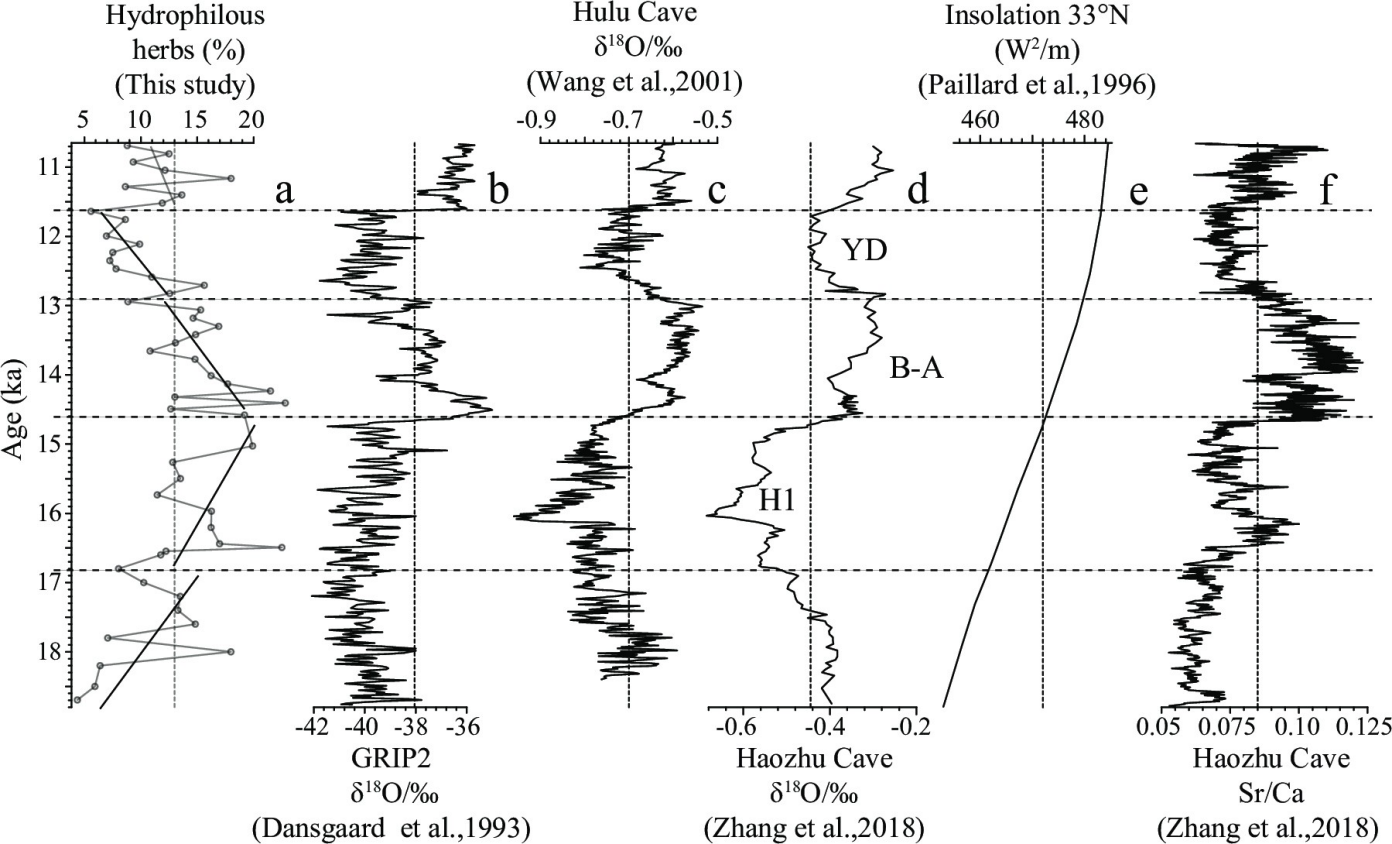
Correlation of (a) the abundance trends of hydrophilous herbs from the Xinmocun section with: (b) the δ^18^O record from GRIP2 [33], (c) the δ^18^O record from Hulu cave stalagmites [11], (d) the δ^18^O record from Haozhu cave stalagmites [10], (e) insolation at 33°N [32], and (f) the Sr/Ca ratio from Haozhu cave stalagmites [10]. For each time interval, the solid line is linear fitting (a).

This period was characterized by a gradual increase in the abundance of hydrophilous herbs [Fig 4A], suggesting that the climate became more humid. In contrast, despite the slow strengthening of insolation during this period [Fig 4E] [32], climate cooling was indicated by a negative δ^18^O shift in the Greenland ice core [Fig 4B] [33] and increased precipitation was implied in stalagmite records from Hulu [Fig 4C] [11] and Haozhu [Fig 4D] [10] caves in China. This cooler climate and increased precipitation was inconsistent with the idea that increased ISM activity was linked to a warmer climate. Instead, it was more consistent with the cold humid climate of the middle Yangtze region inferred from low Sr/Ca ratios in stalagmites from Haozhu cave [Fig 4F] [10]. Accordingly, we consider that global cooling during this period was responsible for increased humidity at the eastern margin of the TP.

### 16.8–14.6 ka

Hydrophilous herbs continued to increase in abundance during this period [Fig 4A]. A positive shift in the δ^18^O record of the Greenland ice core [Fig 4B] [33] and of stalagmites in Hulu [Fig 4C] [11] and Haozhu [Fig 4D] [10] caves indicated a general warming and wetting trend during this period globally and in China, consistent with the continuous increase in insolation [Fig 4E] [32]. This was consistent with the traditional understanding that ISM activity increases with warming and wetting. A reduction in the Sr/Ca ratio of stalagmites from Haozhu cave indicated that climate in the middle Yangtze region became more humid at this time [Fig 4F] [10].

At 16.8 ka, abrupt increases were recorded in the magnetic susceptibility (SUS) of loess at Yuanbo [12], the total organic carbon (TOC) content of a peat core from Hongyuan [14], and the redness of sediments at Lake Qinghai [34]. These changes suggest that 16.8 ka was an important turning point in the Northern Hemisphere climate, widely believed to indicate the start of the last deglaciation. This timing is consistent with a study that reported the last deglaciation started at 18.5 ka and strengthened at ~16.5 ka in the southern TP area [35]. The onset of the Northern Hemisphere deglaciation was induced by an increase in northern summer insolation, which led to an abrupt rise in sea level [36].

### 14.6–12.9 ka

This period is characterized by the greatest abundance of hydrophilous herbs in the entire section, showing maximum values between 14.6 and 14.0 ka, and decreasing from ~14.0 to 12.9 ka [Fig 4A]. Correspondingly, δ^18^O values from the Greenland ice core and cave stalagmite records were relatively high between 14.6 and 14.0 ka. Between 14.0 and 12.9 ka, δ^18^O values from the Greenland ice core show a decrease trend [Fig 4B] [33], whereas δ^18^O values in cave stalagmites showed an increase [Fig 4C and 4D] [10, 11]. These imply that the climate in the study area during 14.6–14.0 ka was the most humid and warmest of the entire section, which was consistent with high temperatures in high- and low-latitude regions of the Northern Hemisphere. However, temperatures decreased in high-latitude regions from 14.0 to 12.9 ka and increased in low-latitude regions. Accordingly, longitudinal temperature gradients increased from 14.0 to 12.9 ka in the Northern Hemisphere and consequently drove the southward retreat of the ISM circulation. We propose that this resulted in a decrease in hydrophilous herbs from 14.0 to 12.9 ka [Fig 4A], accompanied by drought in the middle Yangtze region, although with some fluctuations [Fig 4F]. These trends were linked to a significant increase in insolation during this period [Fig 4E].

### 12.9–11.6 ka

This period corresponds with the Younger Dryas (YD) identified in other paleoclimate records. During this period, hydrophilous herbs decreased to their lowest mean abundance (9.3%) in the entire section [Fig 4A], coeval with a negative shift in the δ^18^O record of the Greenland ice core [Fig 4B] [33] and cave stalagmites [Fig 4C and 4D] [10, 11]. This suggests that the driest climate in the study area was coincident with a cold period in the Northern Hemisphere, in spite of the high and slowly increasing insolation during this time [Fig 4E]. In contrast, the low Sr/Ca ratios of the Haozhu cave stalagmites indicate a humid climate in the middle Yangtze region during this period [Fig 4F] [10] in spite of global cooling during the YD. The high humidity was possibly due to a lengthening of the Meiyu rainy period and shortening of the post-Meiyu stage [10].

### 11.6–10.6 ka

This period is characterized by an abrupt recovery in hydrophilous herbs to typical values of the sequence [Fig 4A], implying a humid climate in the study area. There is also a positive shift in the δ^18^O records of the Greenland ice core [Fig 4B] and cave stalagmites [Fig 4C and 4D], suggesting a general warming and more humid climate [10, 11, 33], which is consistent with the high insolation at this time [32] [Fig 4E]. Such a humid and warm climate is consistent with increased ISM activity. However, the high Sr/Ca ratio of Haozhu cave stalagmites suggests that the climate of the middle Yangtze region was comparatively dry [Fig 4F] [10], possibly related to the high insolation [Fig 4E] [32].

## 6. Conclusion

Pollen analysis of the Xinmocun lacustrine sediments at Maoxian revealed the climate change during the last deglaciation. The results indicate that shrubs and herbs were the dominant vegetation types. Among these, hydrophilous herbs showed a clear change which can be correlated with existing records. The period from 18.7 to 16.8 ka was marked by a cold and humid climate in the study area. During 16.8–14.6 ka, the abundance of hydrophilous herbs increased and δ^18^O records show an increase, indicating a warming climate and increased precipitation in the eastern TP. Between 14.6 and 14.0 ka, the highest abundances of hydrophilous herbs in the entire section correspond to relatively high δ^18^O values. From ~14.0 to 12.9 ka, hydrophilous herbs show a decreasing trend. Intriguingly, longitudinal temperature gradients in the Northern Hemisphere increased during this period and consequently drove the southward retreat of the ISM circulation. This was probably responsible for the decline of hydrophilous herbs in the study area and increased drought in the middle Yangtze region from 14.0 to 12.9 ka. During 12.9–11.6 ka (the YD period), the study area was characterized by a cold and dry climate. Subsequently, hydrophilous herbs show a significant recovery between 11.6 and 10.6 ka, implying a warm and humid climate in the study area. Thus, the ISM began to affect climate in the eastern TP from the onset of the Northern Hemisphere deglaciation at ~16.8 ka.

## Supporting information

S1 Table Two AMS 14C ages of the Xinmocun section at Diexi, Sichuan, East Tibet. (PDF)

S2 Table OSL ages of the Xinmocun section at Diexi, Sichuan, East Tibet. (PDF)

S1 Data (XLSX)
