# Supplementary material for: Pollen record of climate change during the last deglaciation from the eastern Tibetan Plateau
Source: PLoS One. 2020 May 6;15(5):e0232803. doi: 10.1371/journal.pone.0232803 (PMC7202599; doi:10.1371/journal.pone.0232803)
Supplement: S1 Table — (PDF) [file pone.0232803.s001.pdf]

Table 1 Two AMS ^{14}C ages of the Xinmocun section at Diexi, Sichuan, East Tibet.

Beta code	Depth (m)	Material	$\delta^{13}C$ (‰)	Conventional age (a BP)	Calibrated age (cal BC,2σ)	Calibrated age (cal BP,2σ)
421572	6.9	plant	-26.2	12450±50	12955-12305	14905-14255
421571	8.3	plant	-26.3	13640±50	14635-14340	16585-16290
