# Supplementary material for: Pollen record of climate change during the last deglaciation from the eastern Tibetan Plateau
Source: PLoS One. 2020 May 6;15(5):e0232803. doi: 10.1371/journal.pone.0232803 (PMC7202599; doi:10.1371/journal.pone.0232803)
Supplement: S2 Table — (PDF) [file pone.0232803.s002.pdf]

Table 2 OSL ages of the Xinmocun section at Diexi, Sichuan, East Tibet.

Sample	Depth (m)	Water content (%)	α ccunting rate (ks)	K (%)	Numbers	Equivalent does (Gy)	Does rate (Gy ka^{-1})	OSL age (ka)
XM23	0	1.17	11.74 ± 0.25	2.54 ± 0.25	7	56.22 ± 5.27	5.29 ± 0.37	10.63 ± 1.27
XM11	6.05	3.49	11.58 ± 0.26	2.80 ± 0.28	6	74.61 ± 8.40	5.26 ± 0.37	14.19 ± 1.91
XM9	7.05	3.27	11.06 ± 0.32	2.51 ± 0.25	6	72.51 ± 17.55	4.86 ± 0.34	14.91 ± 3.77
XM5	8.95	12.24	11.01 ± 0.32	2.53 ± 0.25	6	72.89 ± 10.59	4.39 ± 0.29	16.60 ± 2.67
XM1	10.95	21.39	11.53 ± 0.18	2.61 ± 0.26	6	77.02 ± 10.71	4.14 ± 0.26	18.60 ± 2.86
